# Environmental Cadmium and Lead Exposures and Hearing Loss in U.S. Adults: The National Health and Nutrition Examination Survey, 1999 to 2004

**DOI:** 10.1289/ehp.1104863

**Published:** 2012-07-31

**Authors:** Yoon-Hyeong Choi, Howard Hu, Bhramar Mukherjee, Josef Miller, Sung Kyun Park

**Affiliations:** 1 Department of Environmental Health Sciences,; 2 Department of Epidemiology, and; 3 Department of Biostatistics, University of Michigan School of Public Health, Ann Arbor, Michigan, USA; 4 Department of Otolaryngology, University of Michigan Medical School, Ann Arbor, Michigan, USA

**Keywords:** cadmium, epidemiology, hearing, lead, NHANES

## Abstract

Background: Although cadmium and lead are known risk factors for hearing loss in animal models, few epidemiologic studies have been conducted on their associations with hearing ability in the general population.

Objectives: We investigated the associations between blood cadmium and lead exposure and hearing loss in the U.S. general population while controlling for noise and other major risk factors contributing to hearing loss.

Methods: We analyzed data from 3,698 U.S. adults 20–69 years of age who had been randomly assigned to the National Health and Nutrition Examination Survey (NHANES) 1999–2004 Audiometry Examination Component. Pure-tone averages (PTA) of hearing thresholds at frequencies of 0.5, 1, 2, and 4 kHz were computed, and hearing loss was defined as a PTA > 25 dB in either ear.

Results: The weighted geometric means of blood cadmium and lead were 0.40 [95% confidence interval (CI): 0.39. 0.42] µg/L and 1.54 (95% CI: 1.49, 1.60) µg/dL, respectively. After adjusting for sociodemographic and clinical risk factors and exposure to occupational and nonoccupational noise, the highest (vs. lowest) quintiles of cadmium and lead were associated with 13.8% (95% CI: 4.6%, 23.8%) and 18.6% (95% CI: 7.4%, 31.1%) increases in PTA, respectively (*p*-trends < 0.05).

Conclusions: Our results suggest that low-level exposure to cadmium and lead found in the general U.S. population may be important risk factors for hearing loss. The findings support efforts to reduce environmental cadmium and lead exposures.

Hearing loss is one of the most common chronic disabling conditions among older adults (Bainbridge et al. 2008). More than 35 million people ≥ 18 years of age suffer from hearing loss in the United States in 2008, and the prevalence of hearing loss tends to increase dramatically with advancing age (Pleis and Lethbridge-Cejku 2006). Loud noise exposure at workplaces (Choi et al. 2012) and from firearms (Agrawal et al. 2009) is a well-established risk factor. A growing body of evidence suggests that exposure to ototoxic environmental and industrial chemicals also may impact the auditory system and lead to hearing loss (Agrawal et al. 2009; Bainbridge et al. 2008).

Experimental studies suggest that lead exposure induces degeneration in the inner ear receptor cells and latency in auditory nerve conduction velocity (Jones et al. 2008; Lasky et al. 1995; Yamamura et al. 1989) and that cadmium exposure causes apoptosis and alters the arrangement of inner ear receptor cells leading to an elevation in auditory thresholds (Kim et al. 2008; Ozcaglar et al. 2001). However, few epidemiologic studies of the association between low-level lead exposure and hearing loss have been conducted in the general population (Park et al. 2010), and there is only one epidemiologic study on cadmium exposure and hearing loss in U.S. adolescents (Shargorodsky et al. 2011).

The aim of this study was to investigate the associations of environmental cadmium and lead exposure with hearing loss in a representative sample of U.S. adults who participated in the National Health and Nutrition Examination Survey (NHANES) 1999–2004, while controlling for important potential confounding factors, including exposure to loud noises at work (occupational), from firearms, and during recreational activities. We also estimated the joint effects of cadmium and lead, as well as effect modification by noise and other potential risk factors.

## Methods

*Study population*. NHANES, which is conducted by the Centers for Disease Control and Prevention (CDC), National Center for Health Statistics (NCHS), is an ongoing series of cross-sectional surveys designed to obtain information from a representative sample of the civilian noninstitutionalized U.S. population. Data are collected through extensive household interviews to obtain information on health risk factors, including health behaviors, personal environment, and lifestyle. In addition, physical examinations and medical history interviews were conducted at a specially equipped mobile examination center (MEC) (CDC 2007a).

In the 1999–2004 NHANES, half of the participants 20–69 years of age were randomly assigned to the Audiometry Examination Component at MEC. Participants who used hearing aids that could not be removed for testing or who could not tolerate test headphones were excluded (CDC 2005). The initial sample size that was eligible for inclusion in the audiometric examination was 5,742 participants: 1,807 in 1999–2000, 2,046 in 2001–2002, and 1,889 in 2003–2004. An additional 479 participants were excluded because of nonresponses or unreliable responses during the audiometric examination.

For the present analysis, we excluded participants with unilateral hearing loss (*n* = 452) and those with missing information on blood cadmium or lead measurements (*n* = 183), longest job-related Occupational Information Network (O*NET) score (*n* = 376), firearm or recreational noise exposure (*n* = 6), or other demographic or hearing-related variables (*n* = 548), leaving a total of 3,698 observations. Compared with the participants with audiometric data who were excluded from our analysis, participants with audiometric data who were included had lower hearing thresholds, were less likely to be classified as having hearing loss, and were more likely to have completed high school and to be non-Hispanic white [see Supplemental Material, Table S1 (http://dx.doi.org/10.1289/ehp.1104863)]. We excluded an additional 76 participants (2.1%) who had hearing thresholds ≤ 0 dB (indicating better-than-normal hearing) from the linear regression models (*n* = 3,622) to better interpret regression results and to avoid adding a constant before log-transformation of hearing thresholds.

NHANES is a publicly available data set, and all participants in NHANES provide written informed consent, consistent with approval by the NCHS Institutional Review Board.

*Audiometric measurement*. Audiometry examinations were conducted in a sound-isolated room by health technicians trained by an audiologist certified by the National Institute for Occupational Safety and Health. Instrumentation for the audiometry component included an audiometer (model AD226; Interacoustics, Assens, Denmark) with standard headphones (model TDH-39) and insert earphones (Etymotic EarTone 3A) (CDC 2005). Pure-tone air conduction hearing thresholds were obtained for both ears at frequencies of 0.5–8 kHz over an intensity range of –10 to 120 dB. Results for examinees who did not respond to at least one frequency were classified as nonresponses (*n* = 476). As an additional measure of the quality of participants’ responses, the 1*-*kHz frequency was tested twice in each ear, and audiograms with ≥ 10 dB difference between the tests were classified as unreliable responses (*n* = 3) (CDC 2005).

We computed the hearing thresholds (decibel) at speech frequencies as a pure-tone average (PTA) of 0.5, 1, 2, and 4 kHz (Agrawal et al. 2009). Hearing loss was defined as a PTA ≥ 25 dB in either ear, consistent with the definition used by the World Health Organization (Ikeda et al. 2009).

*Blood cadmium and lead measurements*. Blood cadmium and lead were measured at the Environmental Health Sciences Laboratory of the CDC, National Center for Environmental Health (NCEH; Atlanta, GA) after confirming the absence of background contamination in all collection and storage materials (CDC 2001a). Cadmium and lead concentrations were measured by a simultaneous multielement atomic absorption spectrometer (SIMAA 6000; PerkinElmer, Norwalk, CT) with Zeeman background correction in NHANES 1999–2002 (CDC 2001a, 2001b) and by an inductively coupled plasma-mass spectrometer (ELAN 6100; PerkinElmer, Shelton, CT) in NHANES 2003–2004 (CDC 2004a).

The detection limit for cadmium was 0.3 µg/L in NHANES 1999–2002 and 0.2 µg/L in NHANES 2003–2004; the detection limit for lead was 0.3 µg/dL in all three NHANES cycles. Of the study participants, 26% and 17% had cadmium concentrations below the detection limit in NHANES 1999–2002 and NHANES 2003–2004, respectively; 0.8% of all participants had blood lead concentrations below the detection limit (CDC 2004a, 2004b, 2007c). For these participants, we imputed a value equal to the detection limit divided by the square root of two (CDC 2007b). Interassay coefficients of variation (CV) ranged from 6.1% to 7.3% and from 4.1% to 4.4% for low and high blood cadmium quality control (QC) pools, and from 4.0% to 7.0% and 3.1% to 3.2% for the low and high blood lead QC pools (CDC 2001a, 2001b, 2004b).

*Noise exposure assessments.* Noise exposures (e.g., occupational, firearm, recreational noise) may be important confounding factors in the associations of blood cadmium and lead with hearing loss. Direct measures of personal noise exposure are not available in the NHANES. Participants were classified as exposed (vs. unexposed) to nonoccupational firearm noise if they indicated that they had ever been exposed to the noise of a firearm at least once a month for 1 year, and were classified as exposed to recreational noise if they indicated exposure to loud noise outside of work (e.g., power tools or loud music) at least once a month for 1 year.

Occupational noise exposures were classified based on occupational noise estimates according to the job title for the longest job held by each participant. These estimates were based on a new occupational noise exposure assessment tool that uses the O*NET survey database (Choi et al. 2012). In brief, the longest job-related O*NET score (range, 1–5) was assigned to each participant as a proxy measure of personal exposure to occupational noise. The median value of the occupational noise score was used to divide participants into low and high occupational noise exposure groups.

*Demographic and hearing-related variables*. Information on other demographic and hearing-related variables was obtained during household interviews or at the MEC. Body mass index (BMI) was calculated as dividing measured weight in kilograms by measured height in meters squared. Use of ototoxic medication was defined based on self-reported use of any aminoglycosides, loop diuretics, antineoplastic drugs, or nonsteroidal anti-inflammatory drugs during the past month. Smoking pack-years were computed, and participants were classified as nonsmokers, smokers with < 20 pack-years, or smokers with ≥ 20 pack-years. Hypertension was classified based on a self-reported physician diagnosis, current use of antihypertensive medication, or systolic blood pressure ≥ 140 mmHg or diastolic blood pressure ≥ 90 mmHg at the time of examination. Diabetes mellitus was classified based on a self-reported physician diagnosis or current use of antihyperglycemic medication, consistent with a previous study of diabetes and hearing loss (Bainbridge et al. 2008).

*Statistical analysis*. All statistical analyses were performed using SAS survey procedures (version 9.2; SAS Institute Inc., Cary, NC) and the R survey package (version 2.9.1; R Foundation for Statistical Computing, Vienna, Austria) to account for the complex survey design and NHANES sample weights (CDC 2007a). We computed 6-year sample weights per NCHS recommendations, which were adjusted for oversampling and nonparticipation of ethnic minorities, the elderly, and low-income persons (CDC 2007a). Two-sided *p*-value < 0.05 was considered statistical significance.

Hearing thresholds for PTA and individual frequencies were log-transformed to normalize distributions, and linear regression was used to model these outcomes. Blood lead and cadmium were log-transformed based on graphical evaluations suggesting improved fit compared with model estimates for nontransformed exposures, although estimates based on fully adjusted natural spline models indicated nonlinear associations with both log-transformed and nontransformed exposure variables [see Supplemental Material, Figure S1 (http://dx.doi.org/10.1289/ehp.1104863)]. Therefore, we also modeled quintiles of blood cadmium and lead to better capture nonlinear relationships. We estimated the percent change in hearing threshold for a doubling of the blood cadmium and blood lead as (e^(ln2 × β)^ –1) × 100%, with 95% confidence intervals (CI) estimated as (e^[ln2 × (β ± 1.96 × SE)]^ –1) × 100%, where β and SE are the estimated regression coefficient and standard error. For quintiles, percent changes were estimated by comparing each of the upper four quintiles to the lowest quintile. *p*-Values for a linear trend were computed by fitting the exposure quintile as an ordinal categorical variable coded using integer values (0–4). Logistic regression was used to estimate odds ratios (ORs) for the hearing loss defined as PTA ≥ 25 dB in one or both ears.

We used sequential models to assess the influence of potential confounders: *a*) model A was adjusted for age and age^2^, sex, race/ethnicity [non-Hispanic white (reference), Mexican American, non-Hispanic black, other], education [< high school (reference), high school, > high school], BMI (continuous), ototoxic medication use (yes/no), cigarette smoking [never smoker (reference), < 20 pack-years, ≥ 20 pack-years], hypertension (yes/no), type 2 diabetes (yes/no), and either blood lead or blood cadmium (for the corresponding cadmium or lead model); *b*) model B further adjusted for occupational noise exposure (O*NET score, continuous); and *c*) model C further adjusted for any nonoccupational firearm noise (yes/no) and any recreational noise (yes/no). We kept hypertension and type 2 diabetes in model A, although these variables could be potential intermediates between lead or cadmium exposure and hearing loss (Bainbridge et al. 2008; Chang et al. 2011), because the effect estimates between the models with and without adjustment for hypertension and diabetes were not much different (data not shown).

We also evaluated fully adjusted associations with log-transformed PTA for subgroups defined by age (20–39, 40–59, ≥ 60 years of age), sex, BMI (< 30, ≥ 30 kg/m^2^), education, ototoxic medication use, cigarette smoking/pack-years, hypertension, diabetes, and occupational (< 2.84, ≥ 2.84 O*NET score), firearm, and recreational noise exposure.

Joint effects of blood cadmium and lead with log-transformed PTA were examined in fully adjusted models using a combined categorical variable classified as low cadmium and low lead (reference), high cadmium and low lead, low cadmium and high lead, or high cadmium and high lead, with high and low categories defined based on median values for all study participants. Departures from additive joint effects [relative excess risk due to interaction (RERI)] and multiplicative joint effects were computed as recommended by Knol and VanderWeele (2012) with the 95% CI for the RERI computed by the delta method (Hosmer and Lemeshow 1992).

## Results

Weighted means ± SE of age and PTA were 42.06 ± 0.28 years and 12.78 ± 0.24 dB, respectively [see Supplemental Material, Table S2 (http://dx.doi.org/10.1289/ehp.1104863)]. Overall, 441 participants (11.9%) had hearing loss (PTA ≥ 25 dB in one or both ears). The age-adjusted geometric means (95% CIs) of blood cadmium and lead in the entire population were 0.40 (95% CI: 0.39, 0.42) µg/L and 1.54 (95% CI: 1.49, 1.60) µg/dL, respectively (Table 1). Compared with participants without hearing loss, those with hearing loss had significantly higher age-adjusted geometric mean blood cadmium (0.46; 95% CI: 0.42, 0.50 µg/L vs. 0.40; 95% CI: 0.38, 0.42 µg/L) and lead (1.72; 95% CI: 1.62, 1.82 µg/dL vs. 1.52; 95% CI: 1.47, 1.58 µg/dL) levels, respectively. Age-adjusted blood cadmium and lead levels differed by race/ethnicity and were higher in participants who were older, less educated, and ever smokers, and in those with high occupational noise exposure, BMI < 30, and no diabetes. Blood lead levels also were higher in men, in participants who did not use ototoxic medications, and among participants who were exposed to firearm noise and among those who were exposed to recreational noise. Blood cadmium levels were higher in women. Blood cadmium and lead levels were highly correlated (Spearman correlation coefficient = 0.12, *p* < 0.001).

**Table 1:** Age-adjusted geometric means (GMs) and 95% CIs of blood cadmium (µg/L) and lead (µg/dL) by participant characteristic (*n* = 3,698).

Characteristic	Participants [*n* (%)]^a^	Blood cadmium^b^		*p*-Value^c^	Blood lead^b^		*p*-Value^c^
Total	3,698	0.40	(0.39, 0.42)	1.54	(1.49, 1.60)
Hearing loss
No	3,257 (88.8)	0.40	(0.38, 0.42)	1.52	(1.47, 1.58)
Yes	441 (11.2)	0.46	(0.42, 0.50)	0.001	1.72	(1.62, 1.82)	< 0.001
Age (years)
20–39	1,650 (44.8)	0.36	(0.34, 0.38)	1.23	(1.18, 1.29)
40–59	1,385 (43.8)	0.44	(0.41, 0.46)	1.75	(1.67, 1.81)
60–69	663 (11.3)	0.45	(0.42, 0.48)	0.007	2.09	(1.98, 2.21)	0.004
Sex
Male	1,729 (48.6)	0.38	(0.36, 0.40)	1.94	(1.87, 2.02)
Female	1,969 (51.4)	0.43	(0.41, 0.45)	< 0.001	1.24	(1.19, 1.31)	< 0.001
BMI (kg/m2)
< 30	2,484 (69.0)	0.42	(0.39, 0.44)	1.61	(1.54, 1.69)
≥ 30	1,214 (31.0)	0.38	(0.36, 0.40)	0.003	1.40	(1.34, 1.47)	< 0.001
Race/ethnicity
Non-Hispanic white	1,827 (72.5)	0.40	(0.38, 0.42)	1.48	(1.42, 1.55)
Non-Hispanic black	750 (10.5)	0.42	(0.39, 0.45)	1.77	(1.68, 1.87)
Mexican American	805 (6.6)	0.39	(0.36, 0.42)	1.81	(1.69, 1.95)
Other	316 (10.4)	0.46	(0.43, 0.49)	0.014	1.60	(1.47, 1.74)	< 0.001
Education
< High school	974 (16.6)	0.55	(0.51, 0.58)	1.99	(1.89, 2.11)
High school	849 (25.1)	0.45	(0.42, 0.48)	1.62	(1.53, 1.71)
> High school	1,875 (58.3)	0.36	(0.34, 0.37)	< 0.001	1.41	(1.35, 1.47)	< 0.001
Ototoxic medication
No	3,132 (84.1)	0.40	(0.39, 0.42)	1.59	(1.53, 1.66)
Yes	566 (15.9)	0.41	(0.37, 0.45)	0.981	1.31	(1.23, 1.39)	< 0.001
Cumulative cigarette pack-years
Never	2,105 (53.7)	0.29	(0.27, 0.30)	1.33	(1.27, 1.40)
< 20	1,183 (33.7)	0.58	(0.54, 0.61)	1.81	(1.73, 1.89)
≥ 20	410 (12.5)	0.68	(0.61, 0.76)	< 0.001	1.89	(1.78, 2.01)	< 0.001
Current diagnosis of hypertension
No	2,713 (76.8)	0.41	(0.39, 0.43)	1.56	(1.50, 1.62)
Yes	985 (23.2)	0.38	(0.36, 0.41)	0.071	1.49	(1.40, 1.59)	0.164
Current diagnosis of diabetes mellitus		
No	3,485 (95.9)	0.41	(0.39, 0.43)	1.56	(1.51, 1.62)
Yes	213 (4.1)	0.32	(0.28, 0.37)	< 0.001	1.20	(1.07, 1.35)	< 0.001
Occupational noise exposure (O*NET score)		
Low (< 2.84)	1,815 (52.7)	0.37	(0.35, 0.38)	1.31	(1.25, 1.38)
High (≥ 2.84)	1,883 (27.3)	0.45	(0.43, 0.48)	< 0.001	1.85	(1.77, 1.92)	< 0.001
Firearm noise exposure
No	3,468 (92.5)	0.40	(0.39, 0.42)	1.52	(1.46, 1.57)
Yes	230 (7.5)	0.41	(0.36, 0.47)	0.872	1.94	(1.72, 2.18)	< 0.001
Reacreational noise exposure
No	2,844 (74.0)	0.40	(0.38, 0.42)	1.47	(1.41, 1.54)
Yes	854 (26.0)	0.42	(0.39, 0.45)	0.184	1.77	(1.67, 1.88)	< 0.001
aWeighted percentages from survey frequency. bAge adjusted except for age groups, which are presented as the unadjusted lead and cadmium levels. cWe used survey t-test for binominal groups and the Wald F-test for categorical groups.												

Table 2 presents the estimated percent changes in PTA in association with blood cadmium and lead levels. Blood cadmium and lead levels, both as continuous variables and when modeled as quintiles, were positively associated with higher (poorer) hearing thresholds in all models. In the fully adjusted model (model C), subjects in the highest blood cadmium quintile had 13.8% (95% CI: 4.6%, 23.8%) higher hearing thresholds than did those in the lowest quintile. A doubling of blood cadmium was associated with a 4.1% (95% CI: 1.2%, 7.1%) increase in hearing thresholds. Similarly, subjects in the highest blood lead quintile had 18.6% (95% CI: 7.4%, 31.1%) higher hearing thresholds than did those in the lowest quintile, and a doubling of blood lead was associated with a 5.4% (95% CI: 2.1%, 8.8%) increase in hearing thresholds. Similar patterns of associations were observed between blood lead and individual frequencies of 0.5, 1, and 4 kHz [see Supplemental Material, Figure S2 (http://dx.doi.org/10.1289/ehp.1104863)].

**Table 2:** Percent change (95% CI) in hearing thresholds (dB) by blood cadmium and lead levels (*n* = 3,622).

Variables	Participants (*n*)	Model A^a^		Model B^b^		Model C^c^	
Cadmium
Per doubling of cadmium	4.38	(1.43, 7.41)	4.07	(1.09, 7.15)	4.13	(1.19, 7.15)
Cadmium quintile (µg/L)
Quintile 1 (0.10–0.20)	1,013	Reference	Reference	Reference
Quintile 2 (0.30–0.30)	553	–1.02	(–8.75, 7.35)	–1.53	(–9.22, 6.81)	–1.22	(–8.86, 7.07)
Quintile 3 (0.40–0.40)	581	2.21	(–5.10, 10.08)	1.26	(–5.95, 9.02)	1.68	(–5.60, 9.53)
Quintile 4 (0.50–0.70)	785	7.07	(–1.07, 15.87)	6.53	(–1.58, 15.32)	6.69	(–1.48, 15.53)
Quintile 5 (0.80–8.50)	690	14.49	(5.17, 24.64)	13.42	(4.18, 23.48)	13.78	(4.55, 23.82)
p-Trend	0.003		0.006		0.005	
Lead
Per doubling of lead	6.24	(2.88, 9.71)	5.68	(2.35, 9.13)	5.41	(2.12, 8.81)
Lead quintile (µg/dL)
Quintile 1 (0.20–0.80)	629	Reference	Reference	Reference
Quintile 2 (0.90–1.30)	842	–0.04	(–9.50, 10.41)	–0.06	(–9.51, 10.38)	–0.50	(–9.94, 9.93)
Quintile 3 (1.40–1.80)	679	7.30	(–3.05, 18.76)	7.11	(–3.23, 18.54)	6.51	(–3.76, 17.89)
Quintile 4 (1.90–2.70)	734	11.86	(0.97, 23.92)	11.01	(0.26, 22.91)	10.22	(–0.40, 21.97)
Quintile 5 (2.80–54.00)	738	21.13	(9.43, 34.09)	19.44	(7.96, 32.14)	18.63	(7.35, 31.09)
p-Trend		< 0.001		< 0.001		< 0.001	
aModel A was adjusted for age, age2, sex, race/ethnicity, education, BMI, ototoxic medication, pack-years of cigarette smoke, hypertension, and diabetes. Cadmium models were further adjusted for lead; lead models were further adjusted for cadmium. bModel B adjusted for all variables included in Model A and further adjusted for occupational noise. cModel C adjusted for all variables in Model B and further adjusted for recreational noise and firearm noise.											

Table 3 shows the logistic regression results for hearing loss in different covariate-adjusted models. Trend tests for blood cadmium levels were significant in all models. Associations with blood lead levels were significant before, but not after, adjusting for noise exposures. Fully adjusted ORs for hearing loss comparing the highest versus the lowest blood cadmium and lead quintiles were 1.7 (95% CI: 1.1, 2.7) and 1.4 (95% CI: 0.8, 2.5), respectively.

**Table 3:** ORs (95% CIs) for hearing lossa by blood cadmium and lead levels (*n* = 3,698).

Variables	No. with hearing loss/ no. of participants	Model A^b^		Model B^c^		Model C^d^	
Cadmium
Per doubling of cadmium	1.28	(1.09, 1.50)	1.26	(1.07, 1.49)	1.26	(1.07, 1.47)
Cadmium quintile (µg/L)
Quintile 1 (0.10–0.20)	71/1,047	Reference		Reference		Reference	
Quintile 2 (0.30–0.30)	53/566	1.20	(0.71, 2.05)	1.17	(0.69, 1.99)	1.21	(0.71, 2.05)
Quintile 3 (0.40–0.40)	72/593	1.07	(0.72, 1.58)	1.01	(0.68, 1.50)	1.05	(0.71, 1.55)
Quintile 4 (0.50–0.70)	128/796	1.44	(0.96, 2.16)	1.39	(0.92, 2.11)	1.39	(0.91, 2.11)
Quintile 5 (0.80–8.50)	117/696	1.80	(1.14, 2.85)	1.72	(1.08, 2.76)	1.74	(1.12, 2.70)
p-Trend	0.009		0.017		0.013	
Lead
Per doubling of lead	1.13	(0.98, 1.30)	1.11	(0.96, 1.28)	1.09	(0.95, 1.26)
Lead quintile (µg/dL)
Quintile 1 (0.20–0.80)	21/659	Reference		Reference		Reference	
Quintile 2 (0.90–1.30)	61/872	1.10	(0.57, 2.10)	1.12	(0.58, 2.15)	1.08	(0.55, 2.12)
Quintile 3 (1.40–1.80)	80/689	1.14	(0.62, 2.09)	1.14	(0.61, 2.11)	1.10	(0.58, 2.05)
Quintile 4 (1.90–2.70)	115/738	1.28	(0.72, 2.27)	1.26	(0.70, 2.27)	1.21	(0.67, 2.22)
Quintile 5 (2.80–54.00)	164/740	1.48	(0.84, 2.62)	1.43	(0.80, 2.57)	1.36	(0.75, 2.48)
p-Trend	0.041		0.084		0.120	
aHearing loss was defined as pure tone average at speech frequencies (0.5, 1, 2, and 4 kHz) > 25 dB. bModel A was adjusted for age, age2, sex, race/ethnicity, education, BMI, ototoxic medication, pack-years of cigarette smoke, hypertension, and diabetes. Cadmium models were further adjusted for lead; lead models were further adjusted for cadmium. cModel B adjusted for all variables included in model A and further adjusted for occupational noise. dModel C adjusted for all variables in model B and further adjusted for recreational noise, and firearm noise.											

We estimated the individual and joint effects of exposures to cadmium and lead on the hearing thresholds (Table 4). Participants with both high cadmium and lead exposures (vs. both low) had a 19.0% (95% CI: 9.7%, 29.1%) increase in PTA that was consistent with additive effects of high cadmium only (7.3%; 95% CI: 0.4%, 14.8%) and of high lead only (10.1%; 95% CI: 0.4%, 20.8%). Estimates did not indicate departures from expectations for additive (RERI = 1.6%; 95% CI: –9.4%, 12.6%; *p* = 0.78) or multiplicative joint effects (percent change of interaction term = 0.7%; 95% CI: –8.9%, 11.39%; *p* = 0.89).

**Table 4:** Percent change (95% CI) in hearing thresholds (dB) by joint effect between blood cadmium and lead levels (*n* = 3,698).

Variables	Low cadmium	High cadmium	Cadmium within strata of lead
Low lead	Reference	7.33 (0.38, 14.77) p = 0.044	7.33 (0.38, 14.77) p = 0.044
High lead	10.09 (0.35, 20.78) p = 0.048	19.01 (9.68, 29.13) p < 0.001	8.1 (–0.87, 17.87) p = 0.085
Lead within strata of cadmium	10.09 (0.35, 20.78) p = 0.048	10.88 (3.40, 18.90) p = 0.006
Measure of interaction on additive scale: RERI = 1.6%; 95% CI: –9.4, 12.6%; p = 0.778. Measure of interaction on multiplicative scale: percent change of interaction term = 0.7%; 95% CI: –8.9, 11.39%; p = 0.891. Models were adjusted for age, age2, sex, race/ethnicity, education, BMI, ototoxic medication, cumulative cigarette pack-years, current diagnosis of hypertension, current diagnosis of diabetes, and occupational, recreational, and firearm noise. Cadmium models were further adjusted for lead, and lead models were further adjusted for cadmium.						

**Figure 1:**
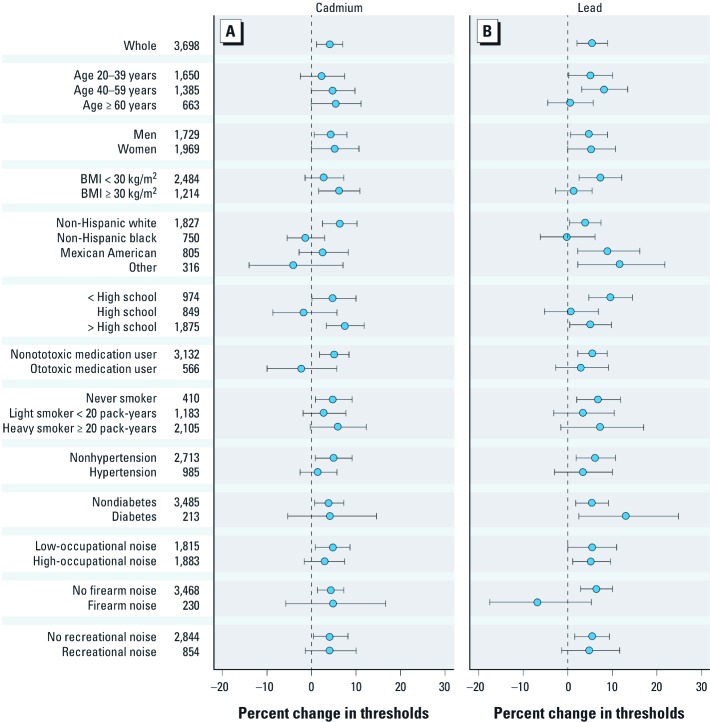
Multivariate-adjusted percent change (95% CI) in hearing thresholds (dB) per doubling of cadmium and lead by participant characterisctic.

Figure 1 presents percent changes in PTA associated with a doubling of lead or cadmium by participant characteristics based on fully adjusted models. For blood cadmium, the association was stronger in non-Hispanic white compared with non-Hispanic black participants (*p =* 0.02). For blood lead, the association was stronger in other race/ethnicity participants than in non-Hispanic whites (*p =* 0.01). There were borderline significant differences in associations between blood lead and PTA when we compared Mexican-Americans with non-Hispanic whites (*p* = 0.05), and participants who were not exposed to firearm noise with those who were exposed (*p =* 0.06).

## Discussion

In a representative sample of U.S. adults who participated in NHANES 1999–2004, environmental cadmium and lead exposures were associated with hearing loss, even after adjusting for socioeconomic factors, noise exposures, and other potential risk factors. In addition, we observed positive dose–response relationships of hearing thresholds with blood cadmium and lead at levels currently observed in the general U.S. population. Compared with the lowest quintiles of cadmium and lead exposure, PTAs for participants in the highest quintiles were increased by 13.8% and 18.6%, respectively. These differences were consistent with estimated differences in PTAs associated with sex (18.4% higher in females vs. males), having diabetes (19.9%), and with a two-unit increase of O*NET occupational noise scores (14.8%, corresponding to the noise exposure difference between “Textile, apparel, and furnishings machine operators” versus “Executive, administrators, and managers” occupation groups) [see Supplemental Material, Table S3 (http://dx.doi.org/10.1289/ehp.1104863)].

Few epidemiologic studies have evaluated associations between low-level lead exposure and hearing outcomes. Our results extend limited evidence from occupational settings or studies of children (Discalzi et al. 1993; Forst et al. 1997; Schwartz and Otto 1991) to the general population. A previous study of 448 elderly community-dwelling men in eastern Massachusetts reported a significant association between hearing loss and an interqurtile range increase in tibia and patella bone lead levels (OR = 1.2; 95% CI: 0.9, 1.5 and OR = 1.5; 95% CI: 1.1, 1.9, respectively) (Park et al. 2010). We estimated an increase in hearing thresholds of 4.1% (95% CI: 1.2%, 7.1%) and OR for hearing loss of 1.1 (95% CI: 1.0, 1.3) with a doubling in blood lead, which extends the body of evidence concerning lead and hearing loss to men and women in the United States as a whole.

To our knowledge, this is the first epidemiologic study to evaluate associations between hearing loss and cadmium exposure in adults. A few experimental studies have suggested possible mechanisms for cadmium ototoxicity. Studies using rats exposed to cadmium-contaminated drinking water suggested that cadmium can induce the generation of reactive oxygen species, loss of mitochondrial membrane depolarization, release of cytochrome c, activation of caspases, apoptosis, and the increase of extracellular signal-regulated kinase activation in auditory cells leading to an elevation in auditory thresholds (Kim et al. 2008; Ozcaglar et al. 2001). A recent study of U.S. adolescents from NHANES 2005–2008 reported a significant association between urinary cadmium and low-frequencies hearing loss (defined as the average of thresholds at 0.5, 1, and 2 kHz > 15dB) (Shargorodsky et al. 2011).

The present study suggests that low-level exposures to cadmium and lead currently observed in the U.S. general population may influence hearing health and supports efforts to reduce environmental cadmium and lead exposures. Compared with participants with exposures in the first quintiles, those in the fifth quintiles of blood cadmium (0.80–8.50 µg/L) and blood lead (2.80–54.0 µg/dL) are at risk for poorer hearing ability. Occupational Safety and Health Administration safety standards are currently 44.5 nmol/L (5 µg/L) for cadmium and 1.93 µmol/L (38.6 µg/dL) for lead in whole blood (Agency for Toxic Substances and Disease Registry 2010a, 2010b). Very few participants in our study population exceeded these limits (0.11% and 0.05% of participants for cadmium and lead standards, respectively); therefore, our findings may at least partly reflect effects on hearing below these levels. Evidence of possible effects of blood lead and cadmium at levels below current standards has also been reported for outcomes such as hypertension, chronic kidney disease, and peripheral arterial disease (Guallar et al. 2006; Navas-Acien et al. 2009; Tellez-Plaza et al. 2008). In the general population, the primary sources of cadmium exposure are cigarette smoke, dietary intake (shellfish, offal, vegetables), and ambient air, particularly in urban, industrial, and contaminated agricultural areas (Järup et al. 1998). Although primary historical sources of lead exposure (gasoline, solder, and paint) have been phased out and environmental lead exposure has decreased considerably in the United States (Hu et al. 2007; Muntner et al. 2005), environmental exposure to low levels of both metals is still widespread (Department of Health and Human Services 2005; Muntner et al. 2005), and their accumulation in the body could influence the development of chronic diseases (Hu et al. 2007; Nordberg et al. 2007).

The present study found a joint effect of combined exposure to high cadmium and high lead on increased hearing thresholds, although formal hypotheses testing for additive and multiplicative interaction was not observed to be significant. The estimated joint effect of lead and cadmium was consistent with additive combined effects of the exposures on hearing resulting in an estimated 19% increase in thresholds in those with high lead and cadmium levels compared with those with low levels of exposure to both metals. Cadmium and lead share several similarities in molecular mechanisms implicated in toxicity. Both metals are divalent cations that interrupt sulfhydryl-containing enzymes, and induce reactive oxygen species increase via oxidation–reduction–inactive metal (Ercal et al. 2001; Vaziri and Khan 2007). They are also associated with changes in intracellular calcium homeostasis (Sabolić 2006), and finally co-exposure to both metals may act synergistically in auditory hair cell death and hearing loss. Few studies have estimated a joint effect of cadmium and lead exposures on other health outcomes. Navas-Acien et al. (2009) investigated the association of lead and cadmium with renal function in > 15,000 adults in the NHANES 1999–2006 and observed a significant interaction between cadmium and lead for albuminuria (*p =* 0.003), based on the model that included the product of the two log-transformed metals. Another study that examined the associations of urinary cadmium and blood lead with reproductive hormones in > 700 young women in the NHANES III also found a stronger estimated effect by both high cadmium and lead in reducing inhibin B level, compared with high lead alone (Gollenberg et al. 2010).

Hypertension and diabetes may be potential causal intermediates rather than confounders because they are risk factors for hearing loss (Bainbridge et al. 2008; Chang et al. 2011) and are health outcomes that may be caused by cadmium and lead exposures (Hu et al. 1996; Schwartz et al. 2003; Tellez-Plaza et al. 2008). Therefore, we compared estimates from models that included all covariates (model A in Tables 2 and 3) except hypertension and diabetes, and found the associations were not changed when hypertension and diabetes were excluded (data not shown).

Another possible explanation for the observed associations of cadmium and lead with shifts in hearing thresholds is that both metal exposures lower bone mineral density (BMD) (Alfvén et al. 2002; Chen et al. 2011) and affect chronic kidney disease (CKD) (Kim et al. 1996; Navas-Acien et al. 2009), which could mediate effects of these metals on hearing loss. This suggestion is supported by several bone disease studies that have observed a high correlation between BMD changes in cochlear capsule and sensorineural hearing loss (Güneri et al. 1996; Monsell et al. 1995) and kidney studies that indicated an association between reduced glomerular filtration rate (an indicator of CKD) and hearing loss (Vilayur et al. 2010).

The important strengths of this study include the use of data from a representative sample of the U.S. general population, which supports generalization of the observed findings; adjustment for potentially important confounding factors, including occupational noise classified using a newly developed assessment tool based on O*NET (Choi et al. 2012), and nonoccupational noise exposures; and the use of NHANES, which uses strict QC procedures.

Several limitations in this study should be considered. The present study was conducted in a cross-sectional design that may raise an issue of validity of causal inferences between lead and cadmium exposures and hearing loss. Blood lead mainly reflects recent exposure, and therefore may not be a good biomarker to predict the long-term effect of low-level lead exposure, such as chronic diseases including hearing loss (Hu et al. 2007). Instead, bone lead has been suggested as a better biomarker of cumulative lead exposure (Hu et al. 2007), and a recent study conducted in eastern Massachusetts showed a significant association between bone lead and age-related hearing loss (Park et al. 2010). Given that the measurement error of exposure is likely to be nondifferential, we would expect that the true association might be larger. Blood cadmium generally reflects current exposure, whereas urinary cadmium is considered cumulative exposure. However, both blood and urinary cadmium are considered the accumulated body burden in general populations with “low” level environmental exposure (Järup et al. 1998). We were not able to examine the association with urinary cadmium because urinary cadmium measures and audiometric tests were conducted in different subsets of NHANES 1999–2004 participants and the number that were included in both subsets is too small for a reliable statistical analysis.

In conclusion, the present study supports the hypothesis that environmental cadmium and lead exposures at levels currently observed in the United States may increase the risk of hearing loss, the third leading chronic condition experienced by adults ≥ 65 years of age (Yueh et al. 2003). Our findings support efforts to reduce environmental cadmium and lead exposures to effectively prevent or delay hearing loss in the general population.

## Supplemental Material

(307 KB) PDF
