# Supplementary material for: Environmental Cadmium and Lead Exposures and Hearing Loss in U.S. Adults: The National Health and Nutrition Examination Survey, 1999 to 2004
Source: Environ Health Perspect. 2012 Jul 31;120(11):1544–50. doi: 10.1289/ehp.1104863 (PMC3556613; doi:10.1289/ehp.1104863)
Supplement: (307 KB) PDF [file ehp.1104863.s001.pdf]

Supplemental Material

Environmental Cadmium and Lead Exposures and Hearing Loss in US Adults: the National Health and Nutrition Examination Survey, 1999 to 2004

Yoon-Hyeong Choi,[1] Howard Hu,[1,2] Bhramar Mukherjee,[3] Josef Miller,[4] Sung Kyun Park[1,2]

[1]Department of Environmental Health Sciences, University of Michigan School of Public Health, Ann Arbor, MI, USA
[2]Department of Epidemiology, University of Michigan School of Public Health, Ann Arbor, MI, USA
[3]Department of Biostatistics, University of Michigan School of Public Health, Ann Arbor, MI, USA
[4]Department of Otolaryngology, University of Michigan Medical School, Ann Arbor, MI, USA

Table of Contents

Supplemental Material, Table S1. General characteristics of study participants and non-participants

Supplemental Material, Table S2. Participant characteristics by hearing loss status

Supplemental Material, Table S3. Percent change (95% CIs) of hearing thresholds (dB) by contribution of different variables in a multiple linear regression

Supplemental Material, Figure S1. Graphical distribution of blood cadmium and lead with log-transformed hearing thresholds

Supplemental Material, Figure S2. Percent change (%) of hearing thresholds (dB) by blood cadmium and lead quintiles at each frequency from 0.5 kHz to 8 kHz

Supplemental Material, Table S1. General characteristics of study participants and non-participants (N=5263[a])

Characteristic	Participants[b] (N=3698)	Non-Participants (N=1565)	*P-value*
Occupation noise exposure[c] *(O*NET score)*	3.06 (± 0.02)	3.12 (± 0.02)[g]	*0.052*
Age *(y)*	42.06 (± 0.28)	42.09 (± 0.43)	*0.956*
Body mass ndex *(wtkg/htm)*	28.04 (± 0.15)	28.47 (± 0.31)[h]	*0.212*
PTA Hearing Thresholds[d] *(dB)*	12.78 (± 0.24)	16.95 (± 0.40)	*<.001*
Hearing Loss[e] *(PTA>25dB, %)*	11.9	28.7	*<.001*
Noise notch[f] *(%)*	17.7	17.0	*0.592*
Sex *(Male %)*	48.6	50.3	*0.275*
Race ethnicity (%)			*<.001*
Non-Hispanic White	72.5	65.7	
Non-Hispanic Black	10.5	11.1	
Mexican American	6.6	9.3	
Other	10.4	13.9	
Education (%)			*<.001*
< High School	16.6	21.8[i]	
High School	25.1	24.5	
> High School	58.3	53.7	
Ototoxic medication *(Current use %)*	15.9	14.8[j]	*0.468*
Cumulative cigarette pack-years (%)			*0.273*
Never	53.7	54.3[k]	
<20	33.7	31.3	
≥20	12.5	14.4	
Hypertension *(%)*	23.2	24.8[l]	*0.289*
Diabetes mellitus *(%)*	4.1	6.4[m]	*8.632*
Firearm noise exposure *(Exposed %)*	7.5	9.2[n]	*0.188*
Reacreation noise exposure *(Exposed %)*	26.0	28.5[o]	*0.126*

Continuous variables: survey t-test, age-adjusted
Categorical variables: 2*2 table or 2*C table : survey X_square (Rao-Scott Chi-Square Test)
[a]Subjects (N=5263) are eligible for the audiometric data
[b]Participants (N=3698) are the individuals having all interest variables in this study: hearing thresholds, hearing loss, age, BMI, sex, race ethnicity, ototoxic medication, cumulative cigarette packyears, hypertension, diabetes mellitus, occupational noise score, and firearm and recreation noise exposure
[c]Occupation noise (1 < O*NET noise scale < 5)
[d]PTA at speech frequencies (Pure tone means at 0.5, 1, 2, 4 KHz)
[e]Hearing loss was defined as PTA at speech frequencies > 25 dB
[f]Noise Notch (Hearing threshold at 3,4, and/or 6 kHz is at least 10 dB greater than at 1 or 2 kHz and at least 10 dB greater than at 6 or 8 kHz.)
Non-Participants (N=1137[g], 1492[h], 1563[i], 1559[j], 1119[k], 1343[l], 1563[m], 1560[n], 1557[o], and 1565 for the others) are the individuals having the variable to be compared

Supplemental Material, Table S2. Participant characteristics by hearing loss status (N=3698[a]).

Characteristic	All participants (N=3698)	Not Hearing Loss (N=3257)	Hearing Loss[a] (N=441)	*P-value*[b]
Blood Lead *(ug/dL)*	1.94 (±0.04)	1.91 (±0.04)	2.17 (±0.09)	*0.006*
Blood Cadmium *(ug/L)*	0.56 (±0.01)	0.55 (±0.01)	0.67 (±0.04)	*0.003*
Age *(y)*	42.06 (±0.28)	40.45 (±0.29)	54.81 (±0.66)	*<.001*
Body mass ndex *(wtkg/htm)*	28.04 (±0.15)	28.03 (±0.15)	28.06 (±0.36)	*0.941*
Pure tone average hearing thresholds (dB)[c]	12.78 (±0.24)	10.98 (±0.16)	27.03 (±0.68)	*<.001*
Occupation noise exposure *(O*NET score)* [d]	3.06 (±0.02)	3.04 (±0.02)	3.25 (±0.04)	*<.001*
Sex *(Male %)*	48.6	46.4	66.3	*<.001*
Race ethnicity (%)				*<.001*
Non-Hispanic White	72.5	71.5	80.6	
Non-Hispanic Black	10.5	11.2	4.5	
Mexican American	6.6	7.1	3.4	
Other	10.4	10.2	11.5	
Education (%)				*0.002*
< High School	16.6	15.1	28.3	
High School	25.1	24.7	28.2	
> High School	58.3	60.2	43.4	
Ototoxic medication *(Current use %)*	15.9	14.8	24.3	*0.002*
Cumulative cigarette pack-years (%)				*<.001*
Never	53.7	55.4	40.8	
<20	33.7	34.6	27.3	
≥20	12.5	10.1	31.9	
Hypertension *(%)*	23.2	20.6	43.7	*<.001*
Diabetes mellitus *(%)*	4.1	3.2	11.4	*<.001*
Firearm noise exposure *(Exposed %)*	7.5	6.8	13.3	*0.001*
Reacreation noise exposure *(Exposed %)*	26.0	25.6	29.3	*0.203*

[a]Hearing loss was defined as pure tone average at speech frequencies > 25 dB.
[b]Survey *t*-test (age-adjusted) for continuous variables and survey (Rao-Scott) Chi-square test for categorical variables were used.
[c]Pure tone average at speech frequencies at 0.5, 1, 2, and 4 kHz.
[d]Occupation noise (1 < O*NET noise scale < 5).

Supplemental Material, Table S3. Percent change (95% CIs) of hearing thresholds (dB) by contribution of different variables in a multiple linear regression

Variables	No.	Regression model[a]
Cadmium Quintile *(ug/L)*		
Quintile 1 (0.10-0.20)	1013	0 (Reference)
Quintile 2 (0.30-0.30)	553	-1.22 (-8.86, 7.07)
Quintile 3 (0.40-0.40)	581	1.68 (-5.60, 9.53)
Quintile 4 (0.50-0.70)	785	6.69 (-1.48, 15.53)
Quintile 5 (0.80-8.50)	690	13.78 (4.55, 23.82)
Lead Quintile *(µg/dL)*		
Quintile 1 (0.20-0.80)	629	0 (Reference)
Quintile 2 (0.90-1.30)	842	-0.5 (-9.94, 9.93)
Quintile 3 (1.40-1.80)	679	6.51 (-3.76, 17.89)
Quintile 4 (1.90-2.70)	734	10.22 (-0.40, 21.97)
Quintile 5 (2.80-54.00)	738	18.63 (7.35, 31.09)
O*NET Noise at longest job *(unit score change)*		7.15 (1.48, 13.13)
Age *(unit year change)*		1.38 (0.05, 2.73)
Age^2 *(unit year change)*		0.01 (0.00, 0.03)
Body mass index *(10 wtkg/htm change)*		1.23 (0.85, 1.61)
Sex		
Male	1729	0 (Reference)
Female	1969	-18.35 (-22.54, -13.94)
Race ethnics		
Non-Hispanic White	1827	0 (Reference)
Non-Hispanic Black	750	-11.88 (-17.96, -5.36)
Mexican American	805	-6.20 (-14.25, 2.60)
Other	316	0.78 (-8.49, 11.00)
Education		
< High School	974	0 (Reference)
High School	849	-9.78 (-16.99, -1.95)
> High School	1875	-19.81 (-24.20, -15.16)
Ototoxic medication		
No	3132	0 (Reference)
Yes	566	2.53 (-4.88, 10.52)
Cumulative cigarette packyears		
Never	2105	0 (Reference)
<20	1183	-1.80 (-8.96, 5.92)
≥20	410	4.46 (-4.45, 14.30)
Current dx of hypertension		
No	2713	0 (Reference)
Yes	985	-1.01 (-5.92, 4.16)
Current dx of diabetes mellitus		
No	3485	0 (Reference)
Yes	213	19.87 (8.93, 31.91)
Noise Exposure at firearm		
No	3468	0 (Reference)
Yes	230	10.19 (-0.52, 22.05)
Noise Exposure at reacreation		
No	2844	0 (Reference)
Yes	854	3.43 (-4.01, 11.46)

[a]Regression model was adjusted for age, age2, bmi, sex, race/ethnicity, ototoxic medication, cumulative cigarette packyears, current dx of hypertension, current dx of diabetes, and ocupation, recreation, and firearm noise. Cadmium models were further adjusted for lead; lead models were further adjusted for cadmium.

Supplemental Material, Figure S1. Graphical distribution of blood cadmium and lead with log-transformed hearing thresholds at speech frequencies (the average of thresholds at 0.5, 1, 2, and 4 kHz)

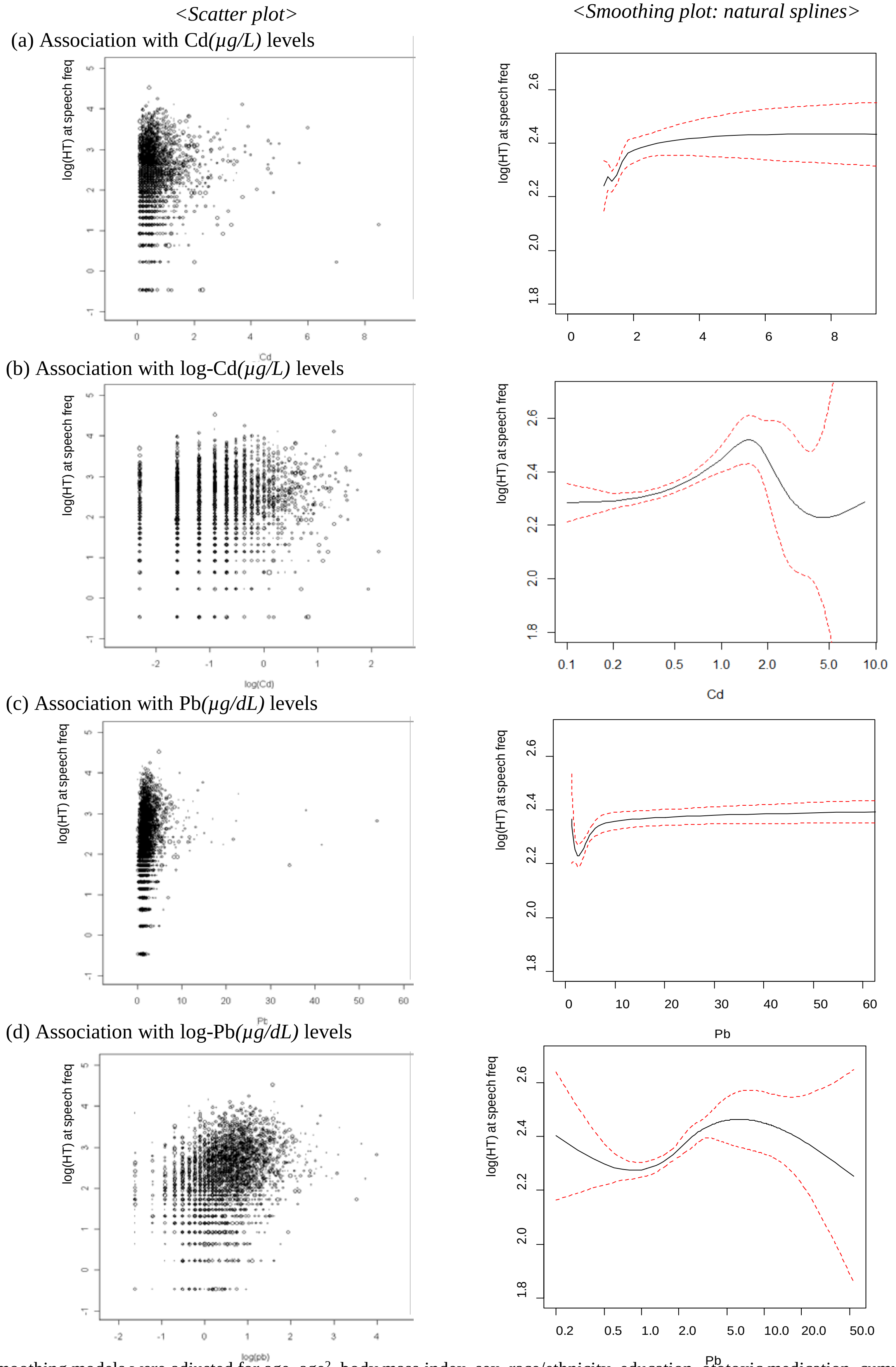

Smoothing models were adjusted for age, age^2, body mass index, sex, race/ethnicity, education, ototoxic medication, cumulative cigarette pack-years, hypertension, diabetes, occupation noise, recreation noise, firearm noise (d.f.=4 , 3 for Cd, Pb; 5 , 4 for log-Cd, log-Pb). Cadmium models were further adjusted for lead; lead models were further adjusted for cadmium.

Supplemental Material, Figure S2. Percent change (%) of hearing thresholds (dB) by blood cadmium and lead quintiles at each frequency from 0.5 kHz to 8 kHz

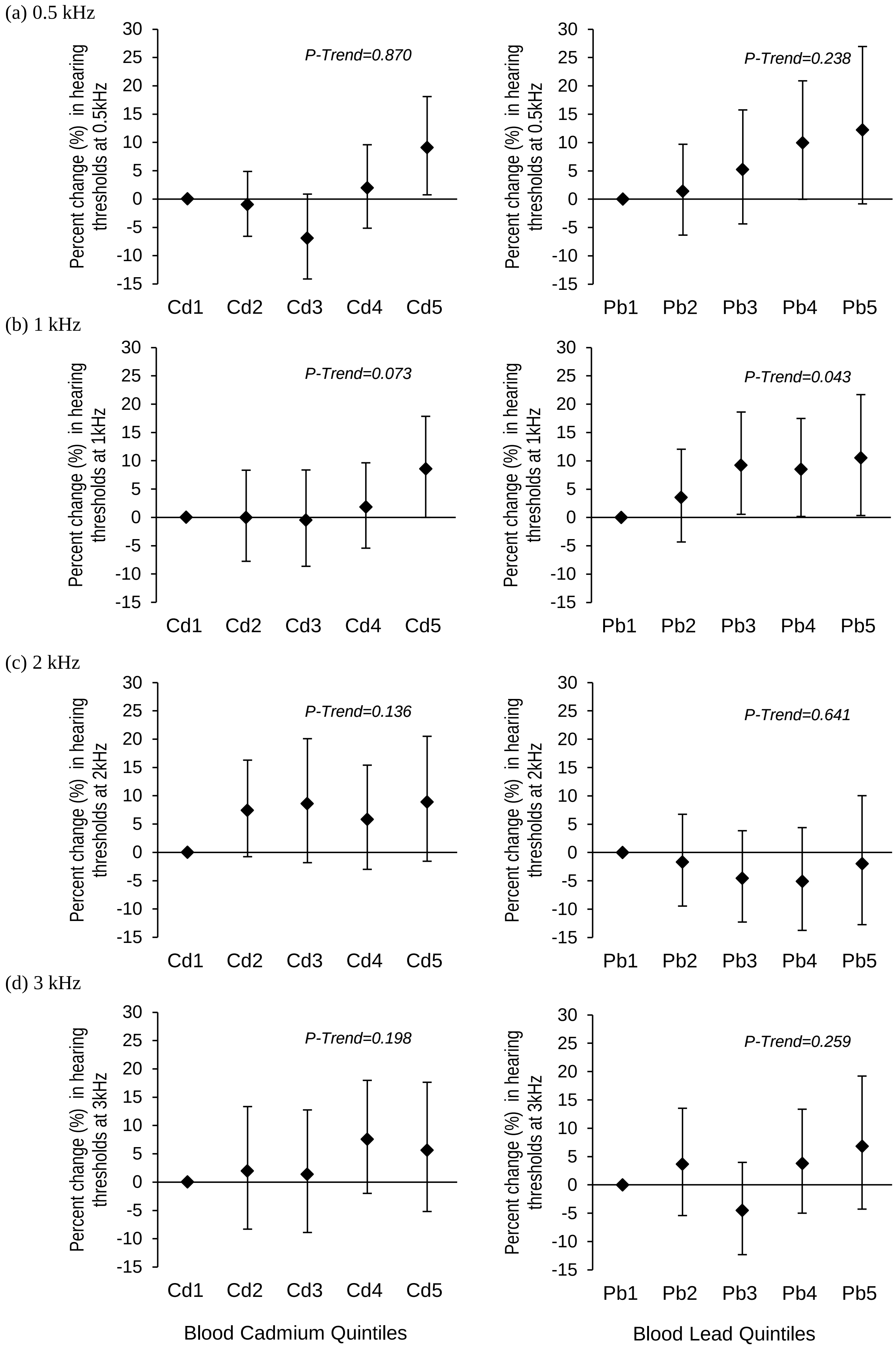

Supplemental Material, Figure S2 (cont.). Percent change (%) of hearing thresholds (dB) by blood cadmium and lead quintiles at each frequency from 0.5 kHz to 8 kHz

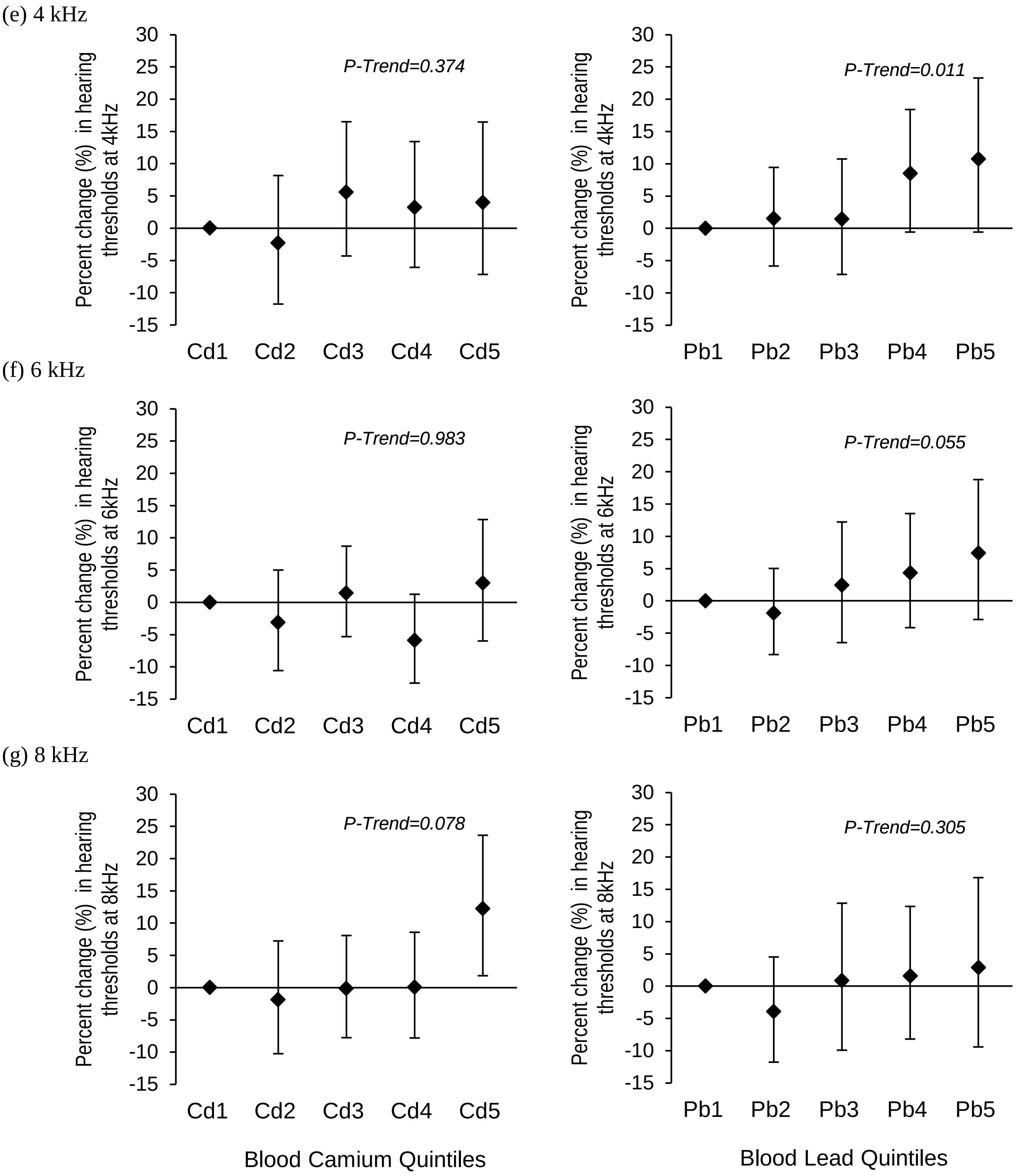

Regression models were adjusted for age, age^2, body mass index, sex, race/ethnicity, education, ototoxic medication, cumulative cigarette pack-years, hypertension, diabetes, occupation noise, recreation noise, firearm noise. Cadmium models were further adjusted for lead; lead models were further adjusted for cadmium.
